# *Achyranthes bidentata* extract exerts osteoprotective effects on steroid-induced osteonecrosis of the femoral head in rats by regulating RANKL/RANK/OPG signaling

**DOI:** 10.1186/s12967-014-0334-7

**Published:** 2014-11-29

**Authors:** Yini Jiang, Yanqiong Zhang, Weiheng Chen, Chunfang Liu, Xiaomin Li, Danni Sun, Zhenli Liu, Ying Xu, Xia Mao, Qiuyan Guo, Na Lin

**Affiliations:** Institute of Chinese Materia Medica, China Academy of Chinese Medical Sciences, No. 16, Nanxiaojie, Dongzhimennei, Beijing, 100700 China; Wangjing Hospital, China Academy of Chinese Medical Sciences, Beijing, 100102 China

**Keywords:** *Achyranthes bidentata* extract, Steroid-induced osteonecrosis, Femoral head, Osteoprotective, RANKL/RANK/OPG signaling pathway

## Abstract

**Background:**

Steroid-induced osteonecrosis of the femoral head (steroid-induced ONFH) presents great challenges due to the various effects of steroids on multi-system pathways involved into osteoblast differentiation, osteoblast and osteoclast apoptosis, lipid metabolism, calcium metabolism and coagulation. As one of the most frequently used herbs in Traditional Chinese Medicine formulas that are prescribed for the regulation of bone and mineral metabolism, the therapeutic effects of Achyranthes bidentata on steroid-induced ONFH remain unclear. Thus, the aim of the current study was to verify whether Achyranthes bidentata extract (ABE) can be used to prevent steroid-induced ONFH and to investigate its underlying pharmacological mechanisms.

**Methods:**

Steroid-induced ONFH rat models were established to evaluate the effects of ABE treatment on osteonecrotic changes and repair processes. Microfocal computed tomography (Micro-CT) was performed to assess the effects of ABE treatment on bone mass, microstructure, and vascularization. Then, the effects of ABE treatment on osteoclast differentiation and bone formation were also evaluated *in vivo* and *in vitro*. In addition, receptor activator of nuclear factor kappa B (RANK), RANK ligand (RANKL), and osteoprotegerin (OPG) expression in sera, femoral heads and bone marrow-derived mesenchymal stem cells (BMSCs) were detected at both protein and mRNA levels.

**Results:**

The ratio of empty lacuna, adipose tissue area, and adipocyte perimeter in the bone marrow were markedly lower in the ABE treatment groups than in the model group. Micro-CT evaluation indicated that ABE treatment could improve the microstructure of the trabecular bone, increase bone mineral density and promote vascularization in steroid-induced ONFH rats. Moreover, ABE treatment inhibited osteoclast differentiation and activated bone formation markers. Interestingly, OPG downregulation, RANK and RANKL upregulation, and an increased ratio of RANKL to OPG in sera and necrotic femoral head could be reversed by ABE treatment, which also effectively inhibited RANKL-induced osteoclast differentiation and regulated RANKL and OPG expression of *in vitro*.

**Conclusion:**

ABE may prevent steroid-induced ONFH and alleviate steroid-induced bone deterioration by regulating the RANKL/RANK/OPG signaling pathway.

## Background

Steroid-induced osteonecrosis of the femoral head (steroid-induced ONFH) is a serious complication in patients who have received steroids for the treatment of various diseases, including nephrotic syndrome, renal transplantation, and systemic lupus erythematosus [1]. As a degenerative bone disease, it leads to the collapse of the femoral head, which subsequently destroys the hip joint and influences the patient’s activities [2]. Owing to the various effects of steroids on multi-system pathways involved in osteoblast differentiation, osteoblast and osteoclast apoptosis, lipid metabolism, calcium metabolism, and coagulation, it has been challenging to fully elucidate the pathogenesis and etiology of steroid-induced ONFH [3]. According to recent studies, ONFH is caused by the impairment of bone cell survival and bone formation, as well as the promotion of osteoclastic resorption and adipocytic differentiation in bone microenvironments [4]. Current treatment for steroid-induced ONFH focuses on preventing irreversible complications, such as biomechanical collapse of the femoral head and osteoarthritis of the hip joint. However, these treatments were limited in their ability to enhance bone repair and to prevent collapse of the articular surface and hip arthroplasty [5]. Therefore, novel and efficient agents for the treatment of this disease are needed.

An increasing number of plant-based therapies derived from traditional Chinese medicine (TCM) have been shown to be effective in the treatment of bone injuries and bone-related diseases via strengthening of bones and muscles and easing joints [6]. *Achyranthes bidentata*, an important medicinal plant of the Amaranthaceae family that has been listed in the Chinese Pharmacopoeia [5], is one of the most frequently used herbs in formulas that are prescribed for the regulation of bone and mineral metabolism [7]. *Achyranthes bidentata* is rich in active phytochemical compounds including oleanolic acid glycosides, saponins, ecdysterone, ketosteroids, and flavonoids, and produces effects that include invigoration of the liver and kidneys, strengthening of the muscles and bones, promotion of blood flow, removal of blood stasis, and increase in longevity [8-10]. Five new oleanolic acid glycosides from *Achyranthes bidentata* have been reported to inhibit the formation of osteoclasts [11]. Of these compounds, ecdysterone and daucosterol markedly stimulate proliferation of osteoblast-like UMR106 cells, and ecdysterone increases osteoblastic activity [12]. The flavonoid quercetin, also found in *Achyranthes bidentata* decreases osteoclastic differentiation [13]. Based on these results, we hypothesize that *Achyranthes bidentata* may produce a therapeutic effect on steroid-induced ONFH. Thus, the aim of the current study was to verify whether *Achyranthes bidentata* extract (ABE) prevents steroid-induced ONFH and to investigate its underlying pharmacological mechanisms.

## Materials and methods

This study was approved by the Research Ethics Committee of the Institute of Chinese Materia Medica, China Academy of Chinese Medical Sciences, Beijing, China. All animals were treated in accordance with the guidelines and regulations for the use and care of animals at the Center for Laboratory Animal Care, China Academy of Chinese Medical Sciences.

### ABE preparation and analysis

*Achyranthes bidentata* was purchased from Beijing Medicinal Herbs Co. Ltd. (Beijing, China), and identified and authenticated by Professor Zhenli Liu at the Institute of Basic Theory of Traditional Chinese Medicine, China Academy of Chinese Medical Sciences. *Achyranthes bidentata* (500 g) was pulverized to a fine powder and boiled twice with 4 L of 80% ethanol for 1 h under reflux. The ethanol extracts were collected and filtered. The filtrates were concentrated under reduced pressure at 50°C to 500 mL by a concentration of 2 g/mL.

**Figure 1:**
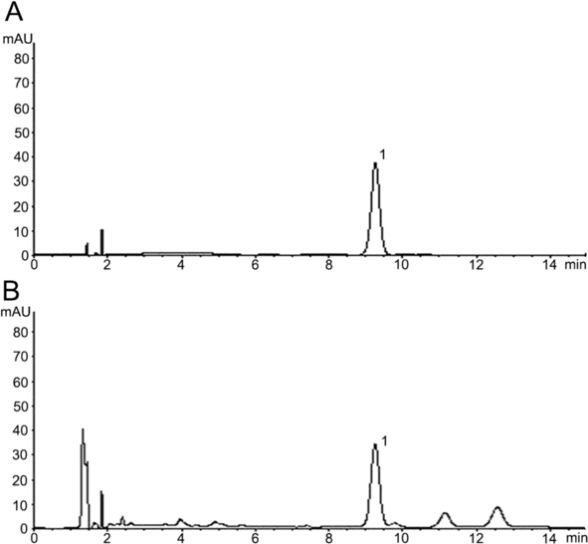
**Chromatograms for β-ecdysone (A) and 80% ethanol extract of** ***Achyranthes bidentata*** **(B).**

An HPLC method was developed for the quantification of β-ecdysone in the extract. A Waters 2695 instrument equipped with a UV detector at 250 nm was used with a stationary phase of Agilent Zorbax SB-C18 (4.6 × 150 mm, 5 μm) at 30°C and a mobile phase of acetonitrile:0.1% methanoic acid (15:85) (Fisher, Waltham, Massachusetts, USA) running at 1 mL/min. The ABE used in this trial contained 0.0425% β-ecdysone (Figure 1).

### Cells and culture system

Bone marrow cells were obtained from the long bones of 4-6-week-old C57 mice (Peking university health science center, Cat No. SCXK: 2011–0012). Bone marrow cells cultured in the presence of M-CSF (20 ng/ml, PeproTech, Inc., Rocky Hill, NJ, USA) for 3 days to generate bone marrow derived macrophages (BMMs).

Bone marrow cells were obtained from the long bones of 4-6-week-old C57 mice (Peking university health science center, Cat No. SCXK: 2011–0012) by flushing with α-minimum essential medium containing antibiotics (Sigma,St. Louis, MO, USA), and further red blood cells (RBC) were removed with RBC lysis buffer (Sigma, St. Louis, MO, USA). The cells were plated on 90-mm culture dishes (Corning, NY, U.S.A.) and incubated for 3 days in a-MEM containing 15% FCS and antibiotics. Adherent cells were used as bone marrow mesenchymal stem cells (BMSCs).

### Animals

One hundred and five 12-week-old male Wistar rats (Cat No. SCXK-(Jun) 2007–004) weighting 300–320 g were obtained from the Experimental Animal Centre of the Academy of Military Medical Sciences (Beijing, China). All rats were maintained in a room equipped with an air-filtering system, and the cages and water were sterilized.

### Groups and treatment

After 1 week of feeding adaptation, the animals were weighed and randomly divided into 5 groups: control (n =20), model (rats with steroid-induced ONFH, n =25), ABE 10 g/kg (rats with steroid-induced ONFH treated with 10 g/kg ABE, n =20), ABE 15 g/kg (rats with steroid-induced ONFH treated with 15 g/kg ABE, n =20), and ABE 22.5 g/kg (rats with steroid-induced ONFH treated with 22.5 g/kg ABE, n =20). The steroid-induced ONFH rat model was implemented according to previous studies [14,15]. Briefly, methylprednisolone acetate (MPSL, Pfizer Manufacturing, Puurs, Belgium) (21 mg/kg) was injected subcutaneously for 6 weeks to induce osteonecrosis. One hour after the MPSL injection, rats in the ABE 10 g/kg, ABE 15 g/kg, and ABE 22.5 g/kg groups, received ABE dissolved in distilled water by oral gavage at 10 g/kg/d, 15 g/kg/d and 22.5 g/kg/d respectively for 6 weeks. In the control and model groups, the rats received no treatment. The animals were fed a standard diet and allowed free activity.

### Tissue sample preparation

Rats from each group were killed 6 weeks after the methylprednisolone injection. The rats were anaesthetized with an intravenous injection of trichloroacetaldehyde hydrate (0.3 mL/kg, Sinopharm Chemical Reagent Co., Ltd, China) and were then killed by exsanguination via an aortectomy. Bilateral femora were obtained at the time of death and the left sides were fixed for 3 days in 4% paraformaldehyde (pH 7.4) to prepare for the microfocal computed tomography (Micro-CT) and light microscopy examinations. After micro-CT scanning, the bone samples were decalcified with ethylenediaminetetraacetic acid (EDTA, 10%, pH 7.4) for 28 days. Samples were sectioned along the coronal plane for the proximal one-third and cut along the axial plane in the distal part (condyle). Finally, the specimens were embedded in paraffin, cut into 5 mm sections, and stained with hematoxylin and eosin. The right sides were stored at −80°C for western blots and real-time PCR.

### Evaluation of steroid-induced ONFH

Osteonecrotic changes and repair processes in steroid-treated rats were observed by histopathological examination using a light microscope 6 weeks after the MPSL injection. The slides were evaluated in a blinded fashion by 3 independent observers. The evaluation criteria for osteonecrosis were based on the report of Yamamoto et al. [16]. Osteonecrosis was judged to be present when there was necrosis of medullary hematopoietic cells or fat cells, empty lacunae, or condensed nuclei in osteocytes. The ratio of empty lacunae (empty lacunae/the total number of osteocytes) was calculated for each femoral head using a coronal section taken at the maximal femoral width. Image Pro 6.0 was used for this calculation.

### Micro-CT

A Micro-CT (μCT, GE Healthcare Biosciences, Piscataway, NJ, USA) was used to detect changes in the excised femoral head sample and bone trabeculae. The following parameters were calculated: bone volume (BV), bone surface (BS), trabecular bone pattern factor (Tb.Pf), structure model index (SMI), trabecular thickness (Tb.Th), trabecular number (Tb.N), trabecular separation (Tb.Sp), and bone mineral density (BMD).

### Quantification and three-dimensional visualization of vessel networks

Femoral head blood vascularization in steroid-treated rats was measured using Micro-CT-based micro-angiography 6 weeks after methylprednisolone injection according to previously reported methods [17,18]. Briefly, rats from each group were anaesthetized as described above and the thoracic and abdomen cavities were opened. A hypodermic needle with disposable infusion device was inserted in the ventriculus sinister with ligation of that proximal to the aorta ascendens. The vasculature was flushed with 500 ml heparinized saline (50 U/ml) at 37°C via a disposable infusion device. After flushing, 500 ml 4% paraformaldehyde solution was pumped into the vasculature to fix the tissues and blood vessels. The vasculature was then injected with Microfil based on the manufacturer’s protocol (Microfil MV-122, Flow Tech, Carver, MA, USA). Animals were then stored overnight at 4°C to ensure polymerization of the contrast agent before microangiography. Bilateral femoral samples were harvested and fixed in 4% paraformaldehyde and 10% EDTA. After perfusion and decalcification, the femoral shaft was fixed in a polymethylmethacrylate sample tube with its long axis perpendicular to the bottom of the tube in preparation for Micro-CT scanning. The scan was perpendicular to the shaft and was initiated from a reference line 10 mm away from the bottom with a scan length of 10 mm.

### Hematological examination

To detect the hyperlipidemia-improving effects of pravastatin, blood samples were collected from the abdominal aorta 6 weeks after methylprednisolone administration. The serum levels of total cholesterol (TC), triglycerides (TG), low-density lipoprotein (LDL), high-density lipoprotein (HDL), apolipoprotein A1 (ApoA1), and apolipoprotein B (ApoB) were determined.

### Analysis of Tartrate-resistant acid phosphatase (TRAP), bone-specific alkaline phosphatase (BAP), receptor activator of nuclear factor kappa B (RANK), RANK ligand (RANKL), and osteoprotegerin (OPG) levels in serum and BMSCs

Serum was separated from 5 mL blood samples. BMSCs were cultured for 3 days in the presence of 15% FCS with or without ABE (0.16, 0.8, 4 μg/ml, respectively) in 96 well culture plate. Supernatants were obtained and stored at −80°C until use. TRAP, BAP, RANK, RANKL, and OPG were measured using enzyme linked immunosorbent assay (ELISA) kits (for TRAP: Kamiya Biomedical Company, Seattle, WA; for BAP: Quidel Corp., San Diego, CA; for RANK, RANKL, and OPG: R&D Systems, Minneapolis, MN) that were specific for the rat. The concentration of the reaction product was determined from a standard curve.

### Western blot analysis

The protein expression levels of RANK, RANKL, and OPG in the femoral head tissues obtained from rats in different groups were detected by western blot analysis. The western blot protocol and semiquantitative analysis were carried out following the protocol of our previous study [19]. The following antibodies were used: RANK antibody (rabbit antibody, dilution 1:50, Cell Signaling Technology, Inc., Danvers, MA, USA), RANKL antibody (rabbit antibody, dilution 1:100, Millipore Corporation, Billerica, MA, USA), OPG antibody (rabbit antibody, dilution 1:100, Santa Cruz Biotechnology, Inc., Santa Cruz, CA, USA), and GAPDH antibody (internal control, rabbit polyclonal antibody, dilution 1:200, Santa Cruz Biotechnology, Inc., Santa Cruz, CA, USA).

### RNA isolation and real-time PCR

The expression of RANK, RANKL, and OPG in the femoral head tissues was analyzed by real-time PCR. A small cube of trabecular bone (proximal femur) was homogenized, and the total RNA was isolated with TRIzol reagent (Invitrogen, Carlsbad, CA, USA). The extracted RNA was dissolved in RNAse-free distilled water. The quality and quantity of the RNA samples were determined by spectrophotometry, with the ratios of absorbance at 260 nm and 280 nm ranging from 1.8 to 2.0. Next, 3 mg of total RNA was reverse-transcribed into cDNA using a High-Capacity cDNA Kit (Takara Bio Inc., Tokyo, Japan) according to the manufacturer’s instructions. The specific transcripts were quantified by quantitative real-time PCR using the QuantiTect SYBR Green PCR Kit (Takara Bio Inc., Tokyo, Japan) and analyzed with an ABI 7500 real-time PCR system (Applied Biosystems, Foster City, CA, USA). Gene-specific primers used for RANK, RANKL, OPG and GAPDH were listed in Table 1. The mRNA levels of RANK, RANKL, and OPG were normalized to GAPDH mRNA levels. PCR was performed as 40 cycles at 94°C for 15 s, 55°C for 30 s, and 72°C for 30 s. The relative mRNA expression was calculated using the comparative CT method.

**Table 1:** **Primer sequences used in this study**

**Primer name**		**Sequence (5’-3’)**
RANK	Forward	GTC TGC AGC TCT TCC CTG AC
	Reverse	GAG GAG CAG GAC GAT GAG AC
RANKL	Forward	ACC AGC ATC AAA ATC CCA AG
	Reverse	TTT GAA AGC CCC AAA GTA CG
OPG	Forward	GTT CTT GCA CAG CTT CAC CA
	Reverse	AAA CAG CCC AGT GAC CAT TC
GAPDH	Forward	ACC CTA AGG CCA ACC GTG AAA AG
	Reverse	CAT GAG GTA GTC TGT CAG GT

### TRAP Staining

TRAP staining was used to identify osteoclasts in vitro and in vivo. To examine osteoclast formation *in vivo*, the osteoclasts number (N.Oc/B.Pm) was expressed using the bone perimeter (B.Pm) as a reference. To examine osteoclast formation *in vitro*, BMMs were resuspended in α-minimum essential medium containing 15% fetal calf serum (FCS), M-CSF (20 ng/ml), RANKL (100 ng/ml, PeproTech) and/or ABE (0.16, 0.8, 4 μg/ml, respectively) in 96 well culture plates (Corning, MA, USA). Six days later, cells were fixed and stained by TRAP staining kit (Sigma Alrich, USA) according to the manufacturer’s protocol. The images were taken with a digital camera attached to the microscope. TRAP positive multinucleated cells (>3 nuclei) were scored as osteoclast-like cells. The number of TRAP-positive cells was counted using an eyepiece graticule at a magnification of 100 and the results expressed as the number of cells per cm^2^.

### Cell viability assay

BMM cells were seeded in 96-well plates and incubated cultured in the presence of M-CSF and RANKL with or without different concentrations of ABE (0.16, 0.8, 4 μg/ml, respectively) for 24 h. BMSCs were cultured with or without different concentrations of ABE (0.16, 0.8, 4 μg/ml, respectively) for 24 h. After drug treatment, cells were washed twice with phosphate-buffered saline (PBS; pH 7.4), and then cell viability was determined by 3-(4,5-Dimethylthiazol-2-yl)-2,5-diphenyltetrazolium bromide (MTT) method using Cell Titer 96® Non-Radioactive Cell Proliferation Assay (Promega, Madison, USA) according to the manufacturer’s instructions. All absorbance at 570 nm were measured with a microplate reader.

### Statistical analysis

SPSS version 13.0 software (SPSS Inc., Chicago, IL, USA) and SAS version 9.1 software (SAS Institute, Cary, NC, USA) were used for statistical analysis. All experiments were performed in triplicate. Continuous variables were expressed as mean ± standard deviation. For comparisons of means among multiple groups, one-way ANOVAs followed by LSD tests were performed. Differences were considered statistically significant when P <0.05.

## Results

### ABE treatment reduces histopathological changes in rats with steroid-induced ONFH

**Figure 2:**
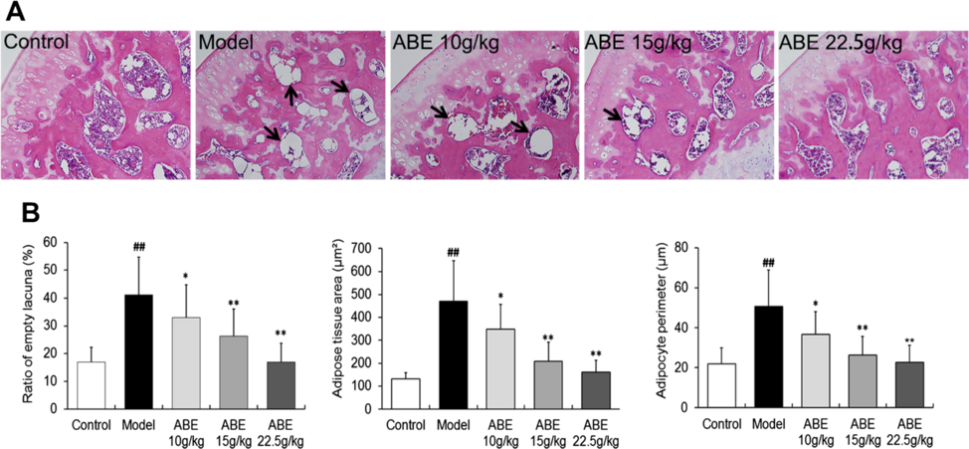
**ABE treatment enhances osteogenesis and reverses bone marrow adipogenesis. (A)** Histological features of normal bone from a normal rat, and osteonecrotic bone from rats with steroid-induced ONFH with or without ABE treatment. **(B)** Statistical analysis of the differences of the ratio of empty lacuna, adipose tissue area, and adipocyte perimeter in the control, model, ABE 10 g/kg, ABE 15 g/kg, and ABE 22.5 g/kg groups. Data are presented as the mean ± S.D. (n =20 for control, n =25 for model group, n =20 for ABE treatment groups). ##: P <0.01, in comparison with the control group. * and **: P <0.05 and P <0.01, respectively, in comparison with the model group. The arrow heads indicate necrosis area.

To evaluate the effect of ABE treatment on steroid-induced ONFH, the osteonecrotic changes and repair processes of rats in each group were histopathologically observed. Compared with the control group, there was an accumulation of bone marrow cell debris found in ONFH lesions in the model group, while ABE treatment dramatically attenuated this change in rats with steroid-induced ONFH (Figure 2A). In addition, the ratio of empty lacunae in the bone trabeculae of the model group was significantly greater than that of the control group (P <0.01, Figure 2A and B), and was decreased by ABE treatment in a dose-dependent manner (Figure 2B). Moreover, the adipose tissue area and adipocyte perimeter in the bone marrow, which were dramatically increased in rats with steroid-induced ONFH, were dose-dependently reduced by ABE treatment (Figure 2B).

### ABE treatment improves the microstructure of the trabecular bone and increases bone mineral density (BMD) in rats with steroid-induced ONFH

**Figure 3:**
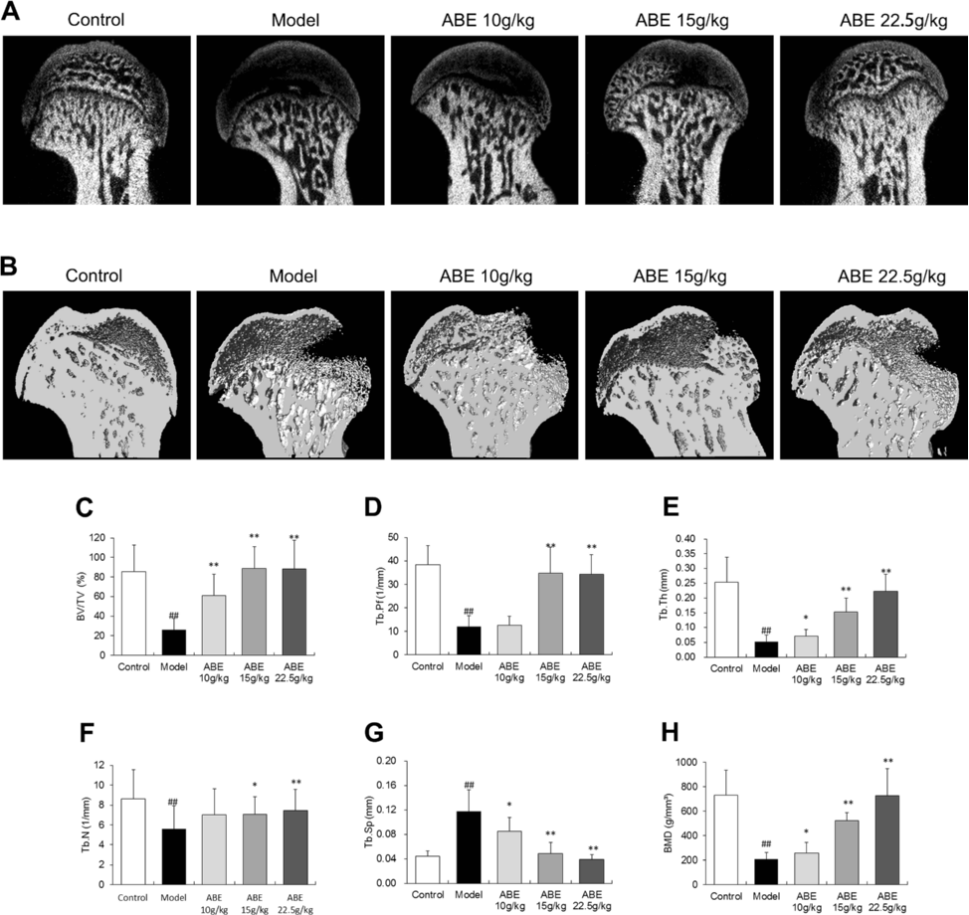
**ABE treatment improves the microstructure of the femoral head. (A)** and **(B)** refer to two-dimensional (2D) and three-dimensional (3D) pictures, respectively, of normal bone from normal rats, and osteonecrotic bone in rats with steroid-induced ONFH with or without ABE treatment. Statistical analysis was performed on the differences in BV/TV **(C)**, Tb.Pf **(D)**, Tb.Th **(E)**, Tb.N **(F)**, Tb.sp **(G)**, and BMD **(H)** in the control, model, ABE 10 g/kg, ABE 15 g/kg and ABE 22.5 g/kg groups. Data are presented as the mean ± S.D. (n =20 for control, n =25 for model, n =20 for ABE 10 g/kg, ABE 15 g/kg and 22.5 g/kg). # and ##: P <0.05 and P <0.01, respectively, in comparison with the control group. * and **: P <0.05 and P <0.01, respectively, in comparison with the model group.

As shown in Figure 3A–G, the bone volume/tissue volume (BV/TV, P <0.01), trabecular thickness (Tb.Th, P <0.01), trabecular bone pattern factor (Tb.Pf), and trabecular number (Tb.N, P <0.01) were significantly reduced, while the trabecular separation (Tb.Sp, P <0.01) and structure model index (SMI, P <0.01) were significantly increased in rats with steroid-induced ONFH when compared with controls. ABE treatment protected rats from steroid-induced effects on the levels of the above microstructural parameters (Figure 3A–G).

To determine whether ABE treatment increases the bone mass of rats with steroid-induced ONFH, BMD values were measured. As shown in Figure 3H, the rats with steroid-induced ONFH showed markedly reduced BMD in the femoral head compared with the control rats (P <0.01). The BMD values in the rats treated with 10.0–22.5 g/kg ABE were increased in a dose-dependent manner compared to those without drug treatment (P <0.01).

### ABE treatment enhances femoral head neovascularization in rats with steroid-induced ONFH

**Figure 4:**
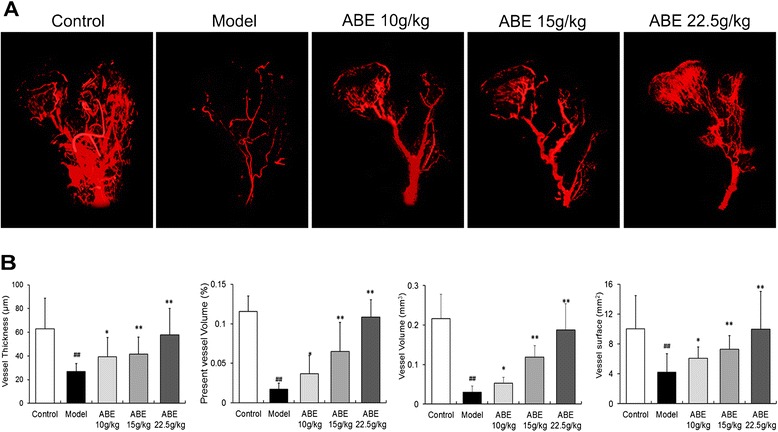
**ABE treatment enhances femoral head neovascularization. (A)** Representative images of micro-CT reconstructed 3-D microangiography of proximal femur from control, model, ABE 10 g/kg, ABE 15 g/kg, and ABE 22.5 g/kg groups. **(B)** Statistical analysis was performed on differences in vessel thickness, percentage of vessel volume, vessel volume, and vessel surface in the femoral heads of rats with steroid-induced ONFH. Data are presented as the mean ± S.D. (n =10). # and ##: P <0.05 and P <0.01, respectively, in comparison with the control group. * and **: P <0.05 and P <0.01, respectively, in comparison with the model group.

The blood vessel microarchitecture of each group was reconstructed in 3 dimensions for presentation. Compared with the control, both the number and the thickness of vessels in necrotic lesions of the femoral head of rats with steroid-induced ONFH were markedly reduced, and vasculatures were not visible, while the samples in the ABE 10 g/kg group showed some capillary vessels, and the samples in the ABE 15–22.5 g/kg groups showed intensive vascular architecture (Figure 4A).

Quantitatively, Figure 4B showed that ABE treatment dose-dependently increased vessel thickness, percent of vessel volume, vessel volume, and vessel surface of the femoral heads of rats with steroid-induced ONFH.

### ABE treatment improves hyperlipidemia in rats with steroid-induced ONFH

**Figure 5:**
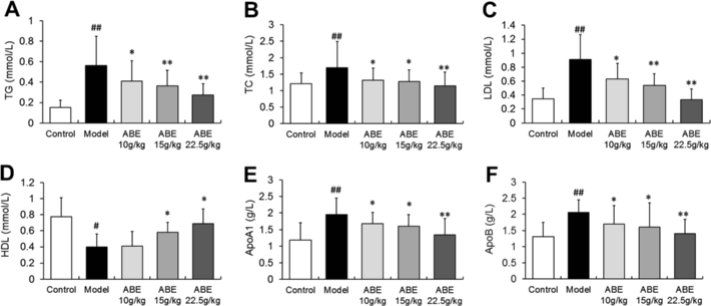
**ABE treatment improves hyperlipidemia in rats with steroid-induced ONFH.** Steroid hormone administration (the model group) induced marked hyperlipidemia, as indicated by significantly elevated TC **(A)**, TG **(B)**, LDL **(C)**, ApoA1 **(E)**, and ApoB **(F)** levels, but significantly decreased HDL levels **(D)**. Doses of 10–22.5 g/kg ABE significantly reduced hyperlipidemia by decreasing TC **(A)**, TG **(B)**, LDL **(C)**, ApoA1 **(E)**, and ApoB **(F)** levels, and increasing HDL levels **(D)**. Data are presented as the mean ± S.D. (n =20 for control, n =25 for model, n =20 for ABE 10 g/kg, ABE 15 g/kg, and 22.5 g/kg groups). # and ##: P <0.05 and P <0.01, respectively, in comparison with the control group. * and **: P <0.05 and P <0.01, respectively, in comparison with the model group.

Blood chemistry data showed that steroid hormone administration (model group) induced marked hyperlipidemia. Steroid administration significantly elevated TG (Figure 5B), TC (Figure 5A), LDL (Figure 5C), ApoA1 (Figure 5E) and ApoB (Figure 5F) levels, but significantly decreased HDL levels (Figure 5D). Administration of 10–22.5 g/kg ABE dose-dependently improved hyperlipidemia by decreasing TG (P <0.01, Figure 5B), TC (P <0.01, Figure 5A), LDL (P <0.05, Figure 5C), ApoA1 (P <0.05, Figure 5E), and ApoB (P <0.05, Figure 5F) levels, and increasing HDL levels (P <0.05, Figure 5D).

### ABE treatment inhibits osteoclast differentiation and activates bone formation markers in rats with steroid-induced ONFH

**Figure 6:**
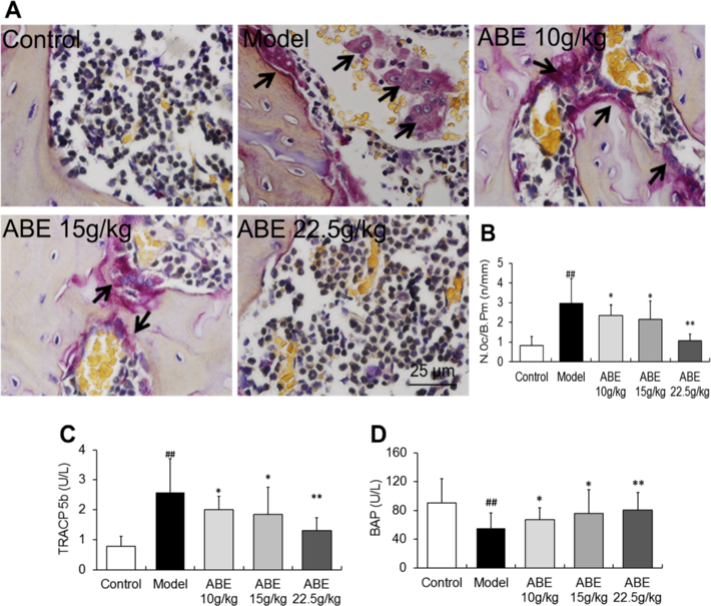
**ABE inhibits osteoclast differentiation in rats with steroid-induced ONFH. (A)** Tartrate-resistant acid phosphatase (TRAP) stained sections from the femoral head of control, model, and ABE-treated rats. **(B)** The number of osteoclasts (multinucleated TRAP-positive cells) in the femoral head of control, model, and ABE-treated rats. **(C)** Serum TRAP activity in the serum of control, model, and ABE-treated rats. **(D)** Serum BAP activity in the serum of control, model, and ABE-treated rats. Data are presented as the mean ± S.D. (n =20 for control, n =25 for model, n =20 for ABE 10 g/kg, ABE 15 g/kg, and 22.5 g/kg groups). # and ##: P <0.05 and P <0.01, respectively, in comparison with the control group. * and **: P <0.05 and P <0.01, respectively, in comparison with the model group. The arrow heads indicate osteoclasts.

To confirm the effect of ABE on the number of osteoclasts, the femoral head sections were stained with TRAP. Only TRAP-positive multinucleated cells located at the bone surface within the bone destruction were considered to be osteoclasts (Figure 6A). Compared with steroid-induced ONFH model rats, the numbers of osteoclasts in the areas of bone destruction were significantly decreased in ABE-treated rats with a tendency for dose-dependence (P <0.01, Figure 6B).

In line with histological observations, serum TRAP activity level was significantly increased in rats with steroid-induced ONFH compared to control rats (0.77 ± 0.34 U/L vs. 2.57 ± 1.13 U/L, P <0.01, Figure 6C). After ABE treatment, serum TRAP activity levels were severely reduced in a dose-dependent manner (Figure 6C). In contrast, serum BAP activity levels decreased to 55.07 ± 21.43 U/L in rats with steroid-induced ONFH, which were significantly lower than control rat levels (90.38 ± 34.12 U/L, P <0.01, Figure 6D). After the ABE treatment, the serum BAP activity level was markedly increased in a dose-dependent manner (Figure 6D).

### ABE treatment regulates RANKL/RANK/OPG signaling in rats with steroid-induced ONFH

**Figure 7:**
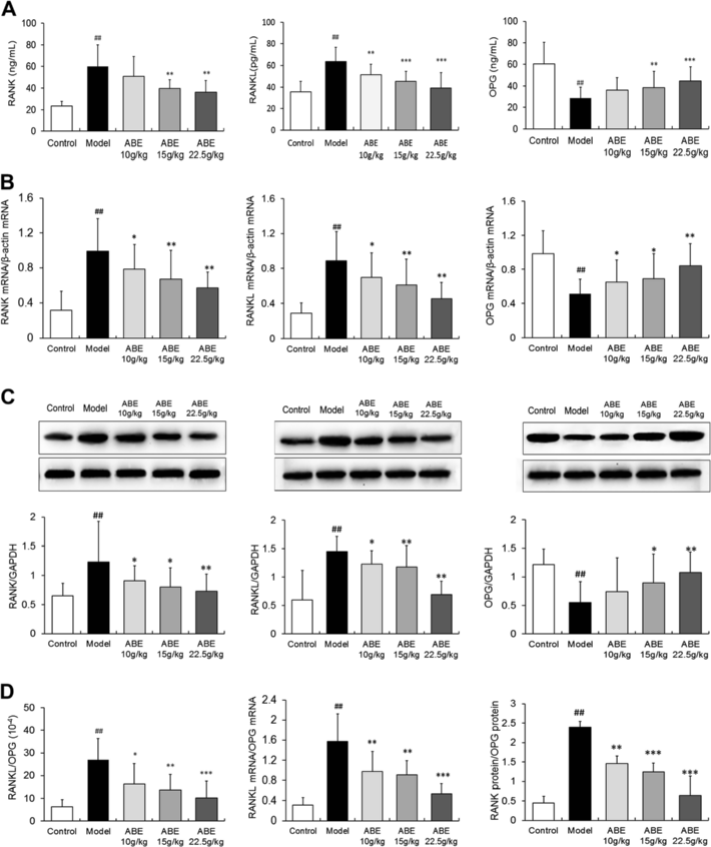
**ABE treatment regulates the RANKL/RANK/OPG signaling pathway in rats with steroid-induced ONFH.** RANK, RANKL, and OPG levels in the serum of rats with steroid-induced ONFH with or without ABE treatment were detected by ELISA **(A)**. RANKL, RANK, and OPG expression in the femoral heads of rats with steroid-induced ONFH with or without ABE treatment were detected at mRNA and protein levels by quantitative real-time RT-PCR **(B)**, western blot **(C)**, respectively. **(D)** shows the ratio of RANKL/OPG in the serum, the ratio of RANKL mRNA/OPG mRNA, and the ratio of RANK protein/OPG protein in the rats with steroid-induced ONFH with and without ABE treatment. Data are presented as the mean ± S.D. (n =20 for control, n =25 for model, n =20 for ABE treatment groups). ##: P <0.01, in comparison with the control group. * and **: P <0.05 and P <0.01, respectively, in comparison with the model group.

We detected changes in RANKL, RANK, and OPG expression at mRNA and protein levels in the sera and femoral heads of rats with steroid-induced ONFH with or without ABE treatment. As shown in Figure 7A, serum RANK and RANKL levels in rats with steroid-induced ONFH were significantly higher than levels in control rats (RANK: 60.32 ± 19.52 U/L vs. 23.33 ± 4.26 U/L, P <0.01; RANKL: 63.33 ± 13.24 U/L vs. 35.74 ± 9.41 U/L, P <0.01). In contrast, serum OPG levels were significantly decreased in rats with steroid-induced ONFH compared with control rats (28.50 ± 10.19 U/L vs. 60.68 ± 19.67 U/L, P <0.01, Figure 7A). After ABE treatment, serum RANK and RANKL levels were dose-dependently reduced, while serum OPG levels were dose-dependently increased (Figure 7A). In addition, ABE treatment significantly reduced transcript abundance of RANKL and RANK, but increased abundance of OPG, in the femoral heads of rats with steroid-induced ONFH (P <0.05, Figure 7B).

In accordance with quantitative real-time RT-PCR results, changes in RANKL, RANK, and OPG protein expression were reversed by 10–22.5 g/kg ABE in a dose-dependent manner (P <0.01, Figure 7C). Furthermore, ABE markedly reduced the ratio of RANKL to OPG in the sera and femoral heads of rats with steroid-induced ONFH in a dose-dependent manner (P <0.01, Figure 7D).

### ABE treatment inhibits RANKL-induced osteoclast differentiation in BMMs

**Figure 8:**
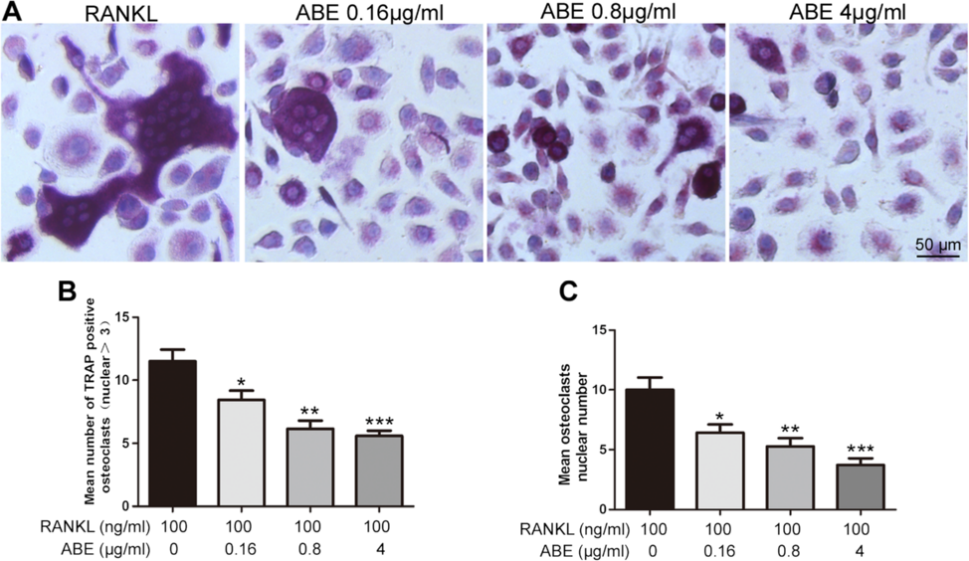
**ABE inhibits RANKL-induced osteoclastdifferentiation inbone marrow derived macrophages (BMMs). (A)** BMMs were cultured in the presence of M-CSF and RANKL withor without different concentrations of ABE (0.16, 0.8, 4 μg/ml, respectively). Six days post-culture, cells were fixed with 4% paraformadehyde followed by TRAP staining. Representative images of TRAP staining of osteoclast-like cells from one of the three experiments are shown. B-C: Quantitative analysis show the mean number of TRAP-positive osteoclast-like cells **(B)**, mean osteoclast-like cells nuclei numbers **(C)**. Data are represented as the mean ± SD of three independent experiments. *P < 0.05, **P < 0.01 and ***P < 0.001 significantly different from Model group.

When BMMs were incubated with M-CSF and RANKL for 6 days, numerous TRAP-positive multi-nucleated osteoclasts were generated (Figure 8A). Addition of ABE (0.16 ~ 4 μg/ml) into the same cultures showed dose-dependent inhibition of osteoclast formation as measured by the TRAP positive multinucleated cells (Figure 8A and B). Notably, osteoclast-like cells in cultures that were treated with ABE exhibited morphological differences from model osteoclast-like cells, containing fewer numbers of nuclei compared to osteoclast-like cells in model (Figure 8C). ABE without the presence of RANKL did not stimulate osteoclast development (data not shown).

### ABE treatment regulates the expression of RANKL and OPG in BMSCs

**Figure 9:**
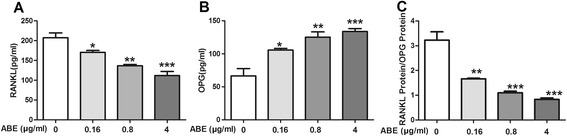
**ABE regulates the expression of RANKL and OPG in bone marrow mesenchymal stem cells (BMSCs).** BMSCs were cultured with or without different concentrations of ABE (0.16, 0.8, 4 μg/ml, respectively). Three days post-culture, supernatants were obtained to detectthe amounts of RANKL **(A)** and OPG **(B)** in the supernatants by ELISA. **(C)** refers to the ratio of RANKL/OPG in the supernatants. Data are represented as the mean ± SD of three independent experiments. *P < 0.05, **P < 0.01 and ***P < 0.001 significantly different from Model group.

**Figure 10:**
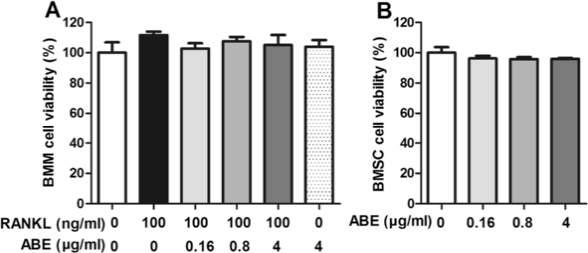
**ABE has no effect on the cell viabilities of bone marrow derived macrophages (BMMs) and bone marrow mesenchymal stem cells (BMSCs). (A)** BMMs were cultured in the presence of M-CSF and RANKL with or without different concentrations of ABE (0.16, 0.8, 4 μg/ml, respectively). **(B)** BMSCs were cultured with or without different concentrations of ABE (0.16, 0.8, 4 μg/ml, respectively). Twenty-four hours later, cells were determined by 3-(4,5-Dimethylthiazol-2-yl)-2,5-diphenyltetrazolium bromide method. Cell viability of the control was taken as 100%. Data are represented as the mean ± SD of three independent experiments.

Compared with model group, doses of 0.16 ~ 4 μg/ml ABE significantly reduced the expression of RANKL, and enhanced the expression of OPG in supernatant of BMSCs with a dose-related manner (Figure 9). More interestingly, ABE treatments markedly decreased the ratio of RANKL to OPG. MTT assay also showed that the anti-osteoclastogenic effect of ABE was not attributable to cellular toxicity (Figure 10).

## Discussion

Excessive steroid treatment induces bone microstructure integrity loss that leads to femoral head collapse and osteoarthritis, and eventually to the need for total hip replacement [20]. Due to the uncertain pathophysiology of steroid-induced ONFH, the best approach to prevent the progression of this disease remains unknown. A large number of kidney-tonifying agents have been used in TCM to treat bone diseases for thousands of years. Among them, ABE has been demonstrated to prevent bone loss [21]. However, its potential role in the treatment of steroid-induced ONFH remains unknown. In the current study, the main findings were: 1) ABE attenuated steroid-induced ONFH by reducing osteonecrotic changes and bone marrow adipogenesis; 2) ABE improved the microstructure of the trabecular bone and increased BMD in the femoral head of rats with steroid-induced ONFH; 3) ABE enhanced femoral head neovascularization and improved the hyperlipidemic state of rats with steroid-induced ONFH; 4) ABE inhibited osteoclast differentiation and improved bone formation by regulating RANKL/RANK/OPG signaling in rats with steroid-induced ONFH; 5) ABE inhibited RANKL-induced osteoclast differentiation in BMMs and regulated the expression of RANKL and OPG in BMSCs.

*Achyranthes bidentata* is an annual herb that is found in hilly districts of China, Korea, Japan, and India, and widely applied in traditional medicine [7]. This plant produces expectorant, anti-inflammatory, antipyretic, antirheumatic, and diuretic effects, and is commonly prescribed for the treatment of spasm, osteodynia of the lumbar region and knees, and flaccidity of limbs [22]. With regard to bone diseases, in 2010 He et al. [23] reported that the *n*-butanol-soluble fraction of *Achyranthes bidentata* root prevented bone loss in ovariectomized rats and may have potential as an alternative treatment for osteoporosis. In 2012, Zhang et al. [21] reported that ABE treatment improved biomechanical bone quality through modification of BMD and trabecular microarchitecture without hyperplastic effects on the uterus, therefore ABE might be a potential alternative treatment for postmenopausal osteoporosis.

The current study was designed to systematically evaluate the therapeutic effects of ABE on steroid-induced ONFH in rats. During histopathological examination, we found that lesions in rats with steroid-induced ONFH showed empty lacunae accompanied by surrounding marrow cell necrosis and occupation of adipocytes, which are primary features of the early stage of this disease. After ABE treatment, the ratio of empty lacunae and the area of bone marrow occupied by adipocytes were significantly reduced, suggesting that improvement in local lipid metabolism was induced by ABE. In addition, the ABE treatment increased bone formation in the femoral head, and these changes were associated with higher bone volume (BV/TV) and trabecular number (TbN). Moreover, ABE improved the trabecular microarchitecture, in part by restoring trabecular connectivity through increasing trabecular thickness (TbTh), while reducing trabecular separation (TbSp), which is consistent with the increase in BMD. These results suggest that ABE could prevent the loss of bone mass induced by excessive steroid treatment.

Because impeded blood flow through the femoral head is implicated in the pathogenesis of steroid-induced ONFH, we applied a novel Micro-CT-based micro-angiography technique to visualize and quantify new blood vessel formation and vascularization in the femoral head of the rat. Recent studies have demonstrated that this technique is quantitative and effective for assessing vascularization [17,18]. Consistent with the improvement produced by ABE treatment on the microstructure of the trabecular bone and BMD, we observed a significant increase in blood vessel volume, vessel surface, percentage of vessel volume, and vessel thickness in the ABE-treated groups, suggesting a dose-dependent increase in vascularization of the femoral heads in this rat model. These findings imply that ABE treatment may produce an environment conducive to bone formation by generating a blood supply for bone reconstruction.

Bone development and maintenance is controlled by the dynamic balance between bone formation by osteoblasts and bone resorption by osteoclasts [24]. The main cause of steroid-induced ONFH is excessive bone resorption that exceeds the rate of bone formation, leading to loss of bone mass. In this context, we observed the effects of ABE treatment on the number of osteoclasts, as well as its regulatory effects on TRAP and BAP, which are markers of osteoblastic bone formation and osteoclastic bone resorption, respectively. Our data showed that ABE treatment reduced numbers of osteoclasts and suppressed serum TRAP, but increased serum BAP, implying that ABE may inhibit osteoclastogenesis while promoting osteoblastogenesis in rats with steroid-induced ONFH.

Accumulating studies have indicated that osteoclast-mediated bone destruction is regulated by the RANKL/RANK/OPG signaling pathway [25,26]. RANK and its ligand RANKL are crucial regulators of osteoclast differentiation. Under physiological conditions, RANKL is expressed in osteoblasts and activated T cells [27], and triggers osteoclast maturation and bone resorption by binding with RANK on osteoclasts. As a soluble decoy receptor for RANKL, OPG is expressed by osteoblasts and inhibits bone resorption by binding with RANKL, which prevents RANKL binding to RANK [28]. Under pathological conditions, the RANKL/RANK/OPG signaling pathway plays a crucial role in the process of bone destruction [29,30]. Bone resorption is regulated locally by the balance between RANKL and OPG. In the current study, we observed down-regulation of OPG, up-regulation of RANK and RANKL, and an increased ratio of RANKL to OPG in the sera and the necrotic femoral head, which were reversed by ABE treatment. Since RANKL is essential and sufficient for the differentiation of osteoclast precursors into mature osteoclasts in the presence of M-CSF [31,32], our data confirmed the inhibitive effects of ABE on RANKL-induced osteoclast formation from BMMs. Moreover, BMSCs and BMMs are originated from bone marrow, and RANKL and OPG expression in BMSCs can regulate osteoclast development and function [33,34]. To clarify the anti-osteoclastogenicaction mechanism of ABE, the RANKL and OPG protein expression levels derived from BMSCs were further detected. As a result, our data showed that ABE treatment could reduce RANKL expression and enhance OPG expression in BMSCs. Thus, both in vivo and in vitro evidence suggest the regulatory effects of ABE treatment on the RANKL/RANK/OPG signaling pathway, which has been considered as a potential target for the prevention of bone destruction in steroid-induced ONFH patients.

In conclusion, our data offer for the first time evidence that ABE prevents steroid-induced ONFH and alleviates steroid-induced bone deterioration by regulating the RANKL/RANK/OPG signaling pathway. Thus, ABE should be considered a potential candidate drug for the treatment of steroid-induced ONFH.
