# Correction to: *Achyranthes bidentata* extract exerts osteoprotective effects on steroid-induced osteonecrosis of the femoral head in rats by regulating RANKL/RANK/OPG signaling

**DOI:** 10.1186/s12967-021-02867-6

**Published:** 2021-05-13

**Authors:** Yini Jiang, Yanqiong Zhang, Weiheng Chen, Chunfang Liu, Xiaomin Li, Danni Sun, Zhenli Liu, Ying Xu, Xia Mao, Qiuyan Guo, Na Lin

**Affiliations:** 1 Institute of Chinese Materia Medica, China Academy of Chinese Medical Sciences, No. 16, Nanxiaojie, Dongzhimennei, Beijing, 100700 China; 2 Wangjing Hospital, China Academy of Chinese Medical Sciences, Beijing, 100102 China

## Correction to: J Transl Med (2014) 12: 334 https://doi.org/10.1186/s12967-014-0334-7

After publication of our article [1], it was brought to our attention that there were errors in three of the figures. These mistakes don’t change the conclusion of this study. The errors are clarified in this Correction article.

## Figure 2

The pathological photograph of the ABE 22.5 g/kg group in Fig. 2a was misplaced. The correct Fig. 2 and its accompanying legend appear below as Fig. 2.

**Fig. 2:**
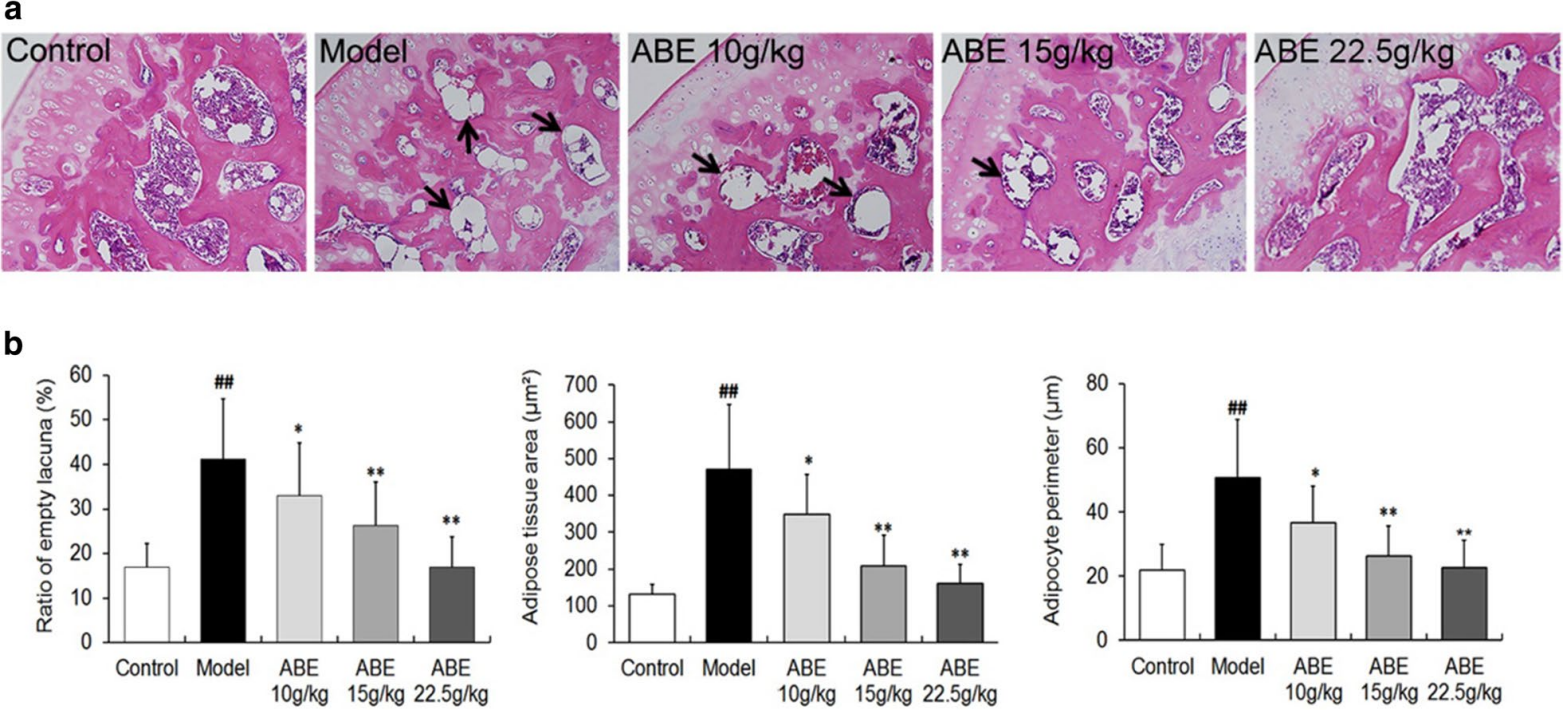
ABE treatment enhances osteogenesis and reverses bone marrow adipogenesis. **a** Histological features of normal bone from a normal rat, and osteonecrotic bone from rats with steroid-induced ONFH with or without ABE treatment. **b** Statistical analysis of the differences of the ratio of empty lacuna, adipose tissue area, and adipocyte perimeter in the control, model, ABE 10 g/kg, ABE 15 g/kg, and ABE 22.5 g/kg groups. Data are presented as the mean ± S.D. (n = 20 for control, n = 25 for model group, n = 20 for ABE treatment groups). ##: P < 0.01, in comparison with the control group. * and **: P < 0.05 and P < 0.01, respectively, in comparison with the model group. The arrow heads indicate necrosis area

## Figure 4

The blood vessel photographs of the ABE 10 g/kg and ABE 15 g/kg group in Fig. 4a was misplaced. The correct Fig. 4 and its accompanying legend appear below as Fig. 4.

**Fig. 4:**
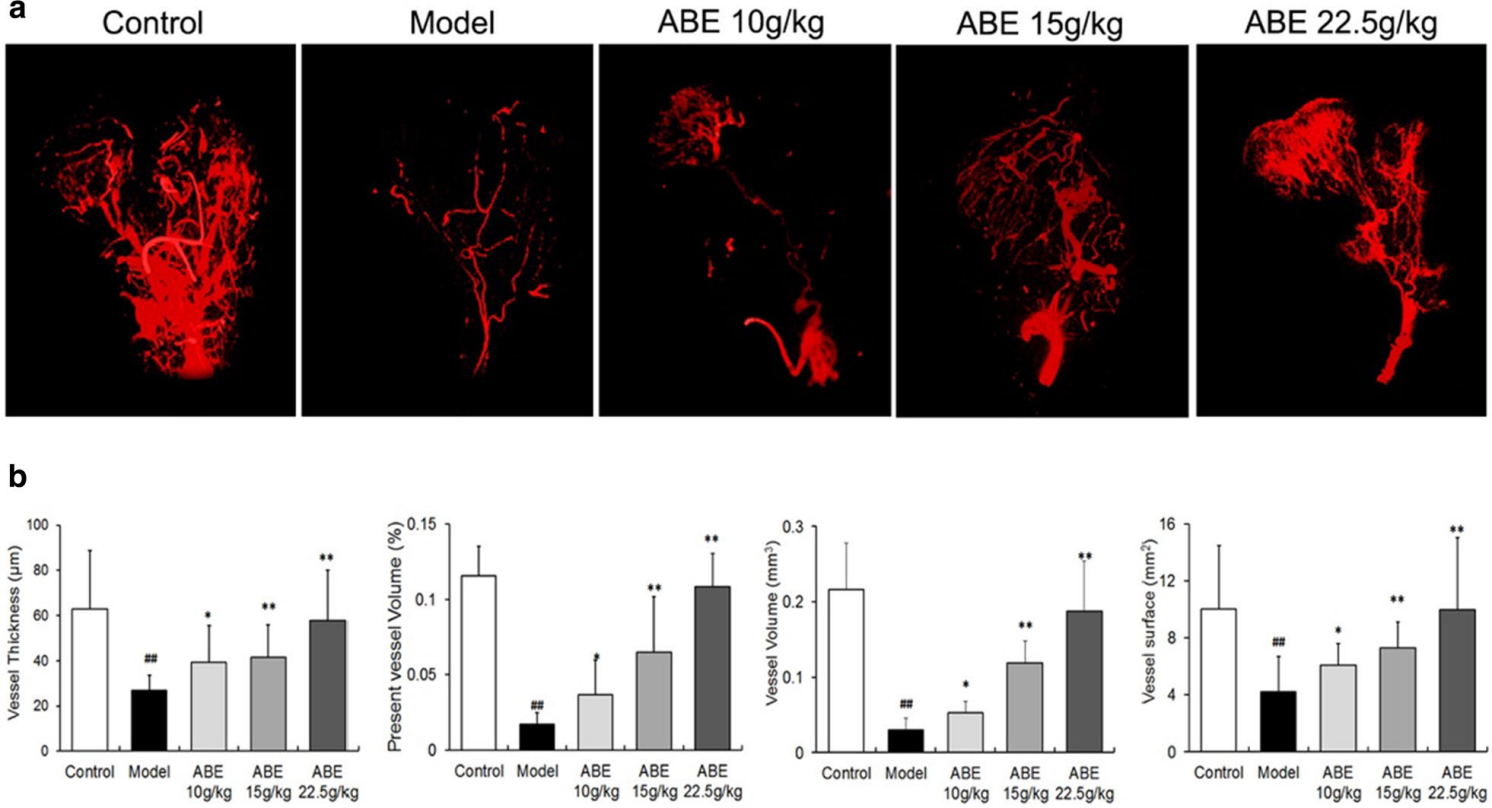
ABE treatment enhances femoral head neovascularization. **a** Representative images of micro-CT reconstructed 3-D microangiography of proximal femur from control, model, ABE 10 g/kg, ABE 15 g/kg, and ABE 22.5 g/kg groups. **b** Statistical analysis was performed on differences in vessel thickness, percentage of vessel volume, vessel volume, and vessel surface in the femoral heads of rats with steroid-induced ONFH. Data are presented as the mean ± S.D. (n = 10). # and ##: P < 0.05 and P < 0.01, respectively, in comparison with the control group. * and **: P < 0.05 and P < 0.01, respectively, in comparison with the model group

## Figure 7

The GAPDH panel photograph of the OPG expression in Fig. 7c was misplaced. The correct Fig. 7 and its accompanying legend appear below as Fig. 7.

**Fig. 7:**
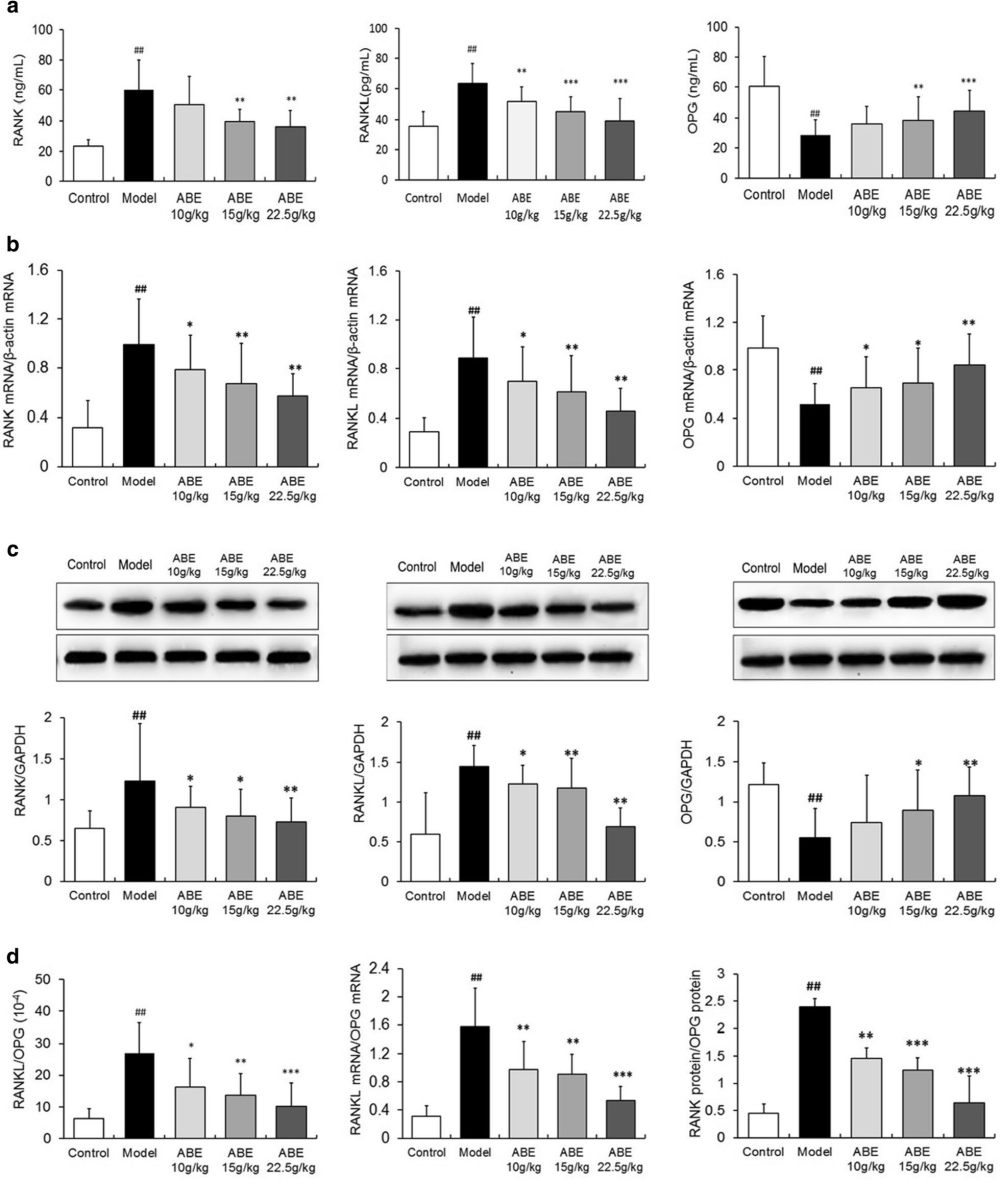
ABE treatment regulates the RANKL/RANK/OPG signaling pathway in rats with steroid-induced ONFH. RANK, RANKL, and OPG levels in the serum of rats with steroid-induced ONFH with or without ABE treatment were detected by ELISA. **a** RANKL, RANK, and OPG expression in the femoral heads of rats with steroid-induced ONFH with or without ABE treatment were detected at mRNA and protein levels by quantitative real-time RT-PCR **b** western blot **c,** respectively. **d** The ratio of RANKL/OPG in the serum, the ratio of RANKL mRNA/OPG mRNA, and the ratio of RANK protein/OPG protein in the rats with steroid-induced ONFH with and without ABE treatment. Data are presented as the mean ± S.D. (n = 20 for control, n = 25 for model, n = 20 for ABE treatment groups). ##: P < 0.01, in comparison with the control group. * and **: P < 0.05 and P < 0.01, respectively, in comparison with the model group
